# Strongyloidiasis Mimicking Carcinoid Syndrome in an Immunocompetent Host

**DOI:** 10.7759/cureus.48608

**Published:** 2023-11-10

**Authors:** Steven Latta, Karine Kasti, Suset Almuinas de Armas, Shiv Krishnaswamy, Andres Reyes-Corcho

**Affiliations:** 1 Internal Medicine, Florida International University, Herbert Wertheim College of Medicine, Miami, USA; 2 Internal Medicine, Memorial Hospital West, Pembroke Pines, USA

**Keywords:** infectious disease medicine, immuno-competent host, carcinoid syndrome, facial flushing, strongyloides stecoralis

## Abstract

Strongyloidiasis is a parasitic infection caused by* Strongyloides stercoralis *which commonly presents as an asymptomatic infection in immunocompetent patients but may cause non-specific gastrointestinal and pulmonary complaints. Here, we report the atypical presentation of strongyloidiasis in a 72-year-old Vietnamese male with shortness of breath and flushing. This case is notable for the unique presentation of cutaneous flushing, the absence of eosinophilia, and negligible microscopic findings on stool examination. Despite insignificant laboratory findings, clinicians should consider strongyloidiasis in patients from endemic areas with unexplained gastrointestinal and pulmonary findings.

## Introduction

Strongyloidiasis is an intestinal nematode infection caused by *Strongyloides stercoralis *that is endemic to Southeast Asia, Africa, and Latin America. Most infections with *S. stercoralis* are asymptomatic but may present with gastrointestinal, pulmonary, or dermatologic symptoms [1]. The spectrum of clinical presentation varies based on the immunological status of the host as well as the chronicity of the parasitic infection. Eosinophilia is often reported as a diagnostic marker in parasitic infections; however, it is only present when the immune regulatory cells react to the parasite or its products [2]. 

Acute infection presents as urticaria or a serpiginous cutaneous reaction where the larvae of *S. stercoralis* penetrate the skin. Once the larvae penetrate the skin, they migrate to the lungs via the circulatory system and make their way to the gastrointestinal system once they are coughed up and swallowed. As the larvae reach the bowel, they cause various gastrointestinal disturbances such as diarrhea, abdominal pain, vomiting, and anorexia. In chronic infections, the lesions appear purpuric and are more commonly found in the perianal region and upper thighs due to the larvae’s ability to migrate faster. Respiratory symptoms such as wheezing and coughing also occur later in the disease course once the larvae migrate to the lungs [2].

When diagnosing *S. stercoralis*, stool microscopy and serological testing are the most cited and reliable tests. Stool microscopy for ova and parasites (O&P) remains the gold standard for diagnosing strongyloidiasis [2]. However, this method has been shown to have a baseline low sensitivity for the detection of *S. stercoralis* due to intermittent excretion of the larvae. For this reason, it is recommended that clinicians obtain numerous stool samples to increase the sensitivity of the test and isolate *S. stercoralis* within the samples. On the other hand, serological testing through enzyme-linked immunoassay (ELISA) detects immunoglobulin linked to specific filariform larval antigens. ELISA has a much higher sensitivity than stool microscopy and is generally considered a strong alternative option [3]. 

## Case presentation

A 72-year-old male with a past medical history of coronary artery disease, type II diabetes mellitus, gastroesophageal reflux disease, and chronic kidney disease was admitted with shortness of breath, chills, and feeling feverish. Upon presentation, the patient was hypertensive with a blood pressure of 154/95 and afebrile. On physical exam, he had a tender and distended abdomen. The patient did not have any guarding or rigidity. The patient’s history was notable for over five prior admissions for diarrhea secondary to *Clostridium difficile* over the past year, and a recent trip to rural Vietnam four months prior. 

**Figure 1:**
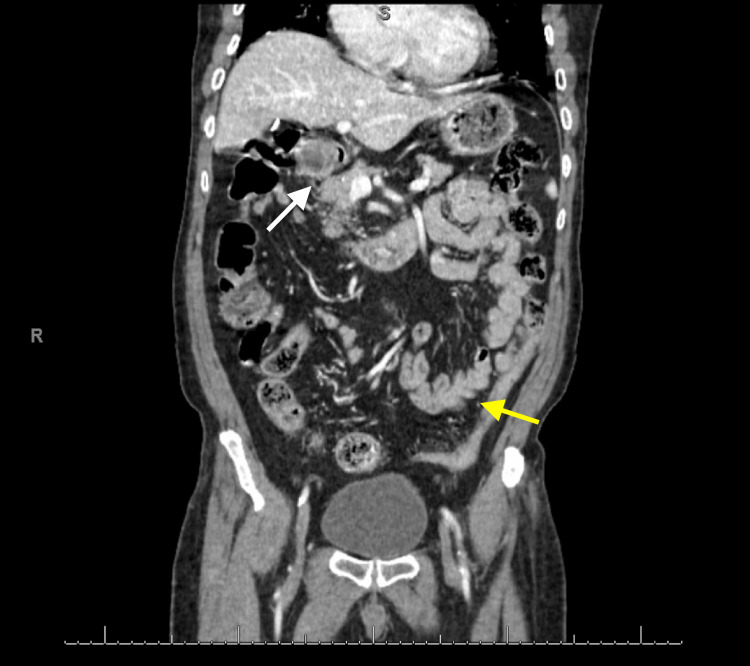
Initial CT scan on admission indicating distal colitis and gastroduodenitis. The white arrow indicates gastroduodenitis and the yellow arrow indicates the distal colitis.

For the past couple of months, the patient reported intermittent headaches, chills, cutaneous flushing, vomiting, watery diarrhea, and a 20 lbs weight loss. At present, the patient is complaining of shortness of breath, chills, and feeling feverish. Initial testing was remarkable for mildly elevated procalcitonin (0.6), mild bilateral pleural effusions, latent Tuberculosis infection, and a CT abdomen/pelvis indicative of distal colitis and gastroduodenitis that can be seen in Figure 1. Complete blood count, erythrocyte sedimentation rate, C-reactive protein, and antinuclear antibody were all within normal limits.

Empiric treatment with cefepime, azithromycin, and metronidazole for seven days was administered but the patient experienced no improvement in his symptoms. A stool O&P, fecal occult blood test, *C. difficile* panel, and testing for malaria were obtained but yielded insignificant findings. The patient remained symptomatic during this period and on day six of admission the patient reported five episodes of watery diarrhea overnight. A more in-depth workup was required, and the patient elected to undergo esophagogastroduodenoscopy and colonoscopy 12 days after admission. Multiple biopsies were taken during this procedure and testing for *Heliobacter pylori*, microscopic colitis, celiac disease, and Whipple’s disease were negative.

**Figure 2:**
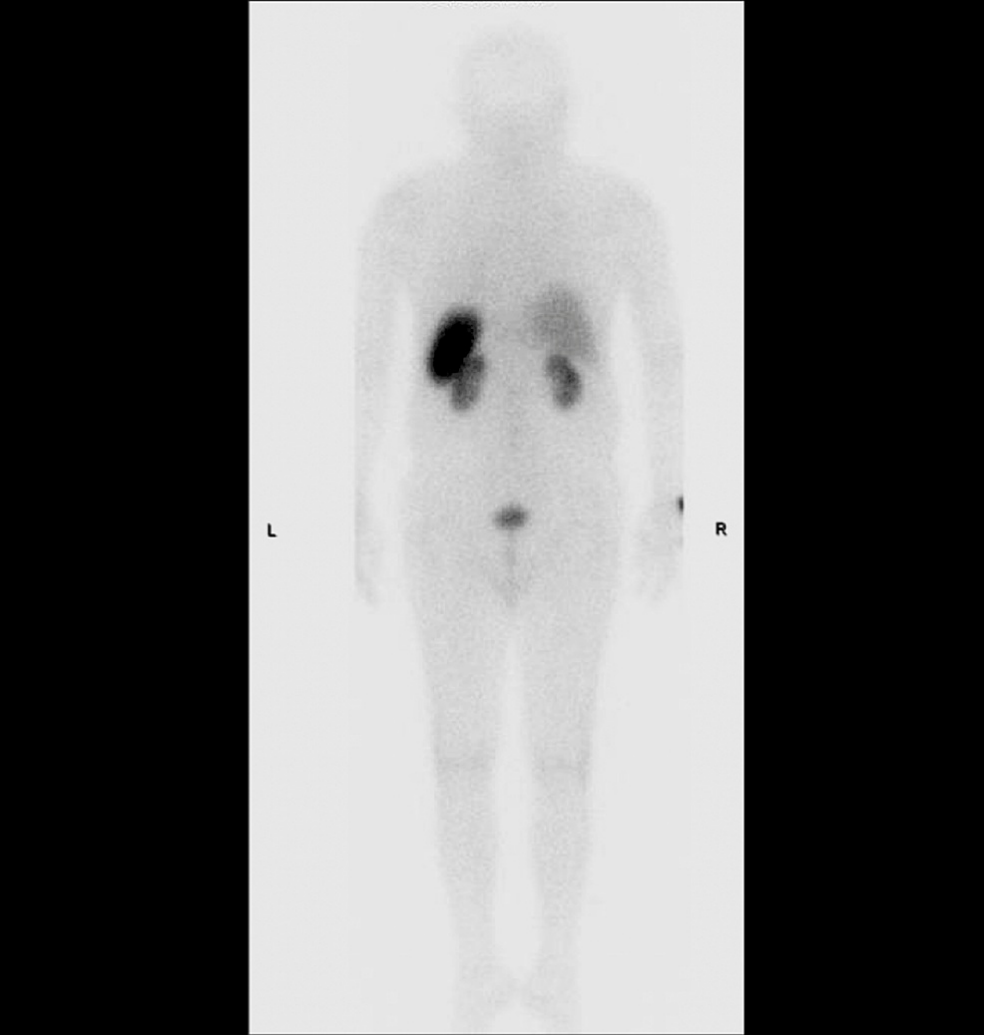
Octreotide scan demonstrating the absence of somatostatin-positive tumors.

At this point, the differential diagnoses included carcinoid syndrome and helminthic infection due to the continued flushing, diarrhea, and shortness of breath experienced by the patient. The workup for a neuroendocrine tumor included urine studies for 5-hydroxyindoleacetic acid (5-HIAA) and an octreotide scan that can be seen in Figure 2. Both tests for carcinoid syndrome were unrevealing and thus the focus shifted toward a helminth infection. Serum studies for multiple helminthic infections were ordered and studies for *Strongyloides* came back positive. The patient was subsequently treated with two doses of oral ivermectin and reported the resolution of his symptoms over the next few days.

## Discussion

While strongyloidiasis is sporadically reported in the United States, infections affect anywhere from 30 to 100 million people worldwide. Infection occurs after the filariform larvae penetrates the skin where it can use blood vessels to migrate to the lungs. From there, the larvae advance through the adjacent pharynx and enter the gastrointestinal tract where they may reproduce. The parasite is later reabsorbed by the duodenal mucosa allowing autoinfection to occur [4]. Autoinfection is defined as a state of dormancy that could allow re-infection by the parasite. It allows the infection to survive in the host for up to 50 years, as the immune system is usually unable to eradicate the parasite [5,6]. Clinical findings vary depending on the parasitic load, and roughly half of immunocompetent patients remain asymptomatic during infection. The most common symptoms are abdominal pain, diarrhea, urticarial rash, and respiratory symptoms [4,5,7]. Hyperinfection syndrome is an uncommon sequela of strongyloidiasis that may present in some patients as shock, disseminated intravascular coagulation, meningitis, and/or respiratory failure, and it is often associated with high mortality rates. Patients with human immunodeficiency virus, human T-cell lymphotropic virus type 1, recent steroid use, or other conditions that impair cell-mediated immunity are at increased risk [8]. 

The diagnosis of *S. stercoralis* requires a high degree of clinical suspicion due to the lack of validated testing. Eosinophilia is often reported as a diagnostic marker in parasitic infections but is only present when the immune regulatory cells react to the parasite or its products. Previous studies have reported only 70% of *S. stercoralis* infections present with eosinophilia, creating a diagnostic challenge for the remaining 30% of cases [2,7]. For direct parasitological detection, a single stool examination has limited sensitivity to diagnose chronic *Strongyloides* [9]. A study from Tan et al. reported a sensitivity of only 25% with this method due to low parasitic load and irregular shedding of larvae [2]. For this method to have clinical utility, clinicians should obtain three to seven stool samples on consecutive days [9]. Due to these diagnostic challenges, serological assays have arrived as alternative strategies for diagnosing *S. stercoralis*. A recent meta-analysis of 19 studies and 3,419 cases from Kalantari et al. reported a sensitivity of 71.7% and a specificity of 89.9% when using serologic studies to diagnose *Strongyloides* [10].

In this case, the patient's primary symptoms comprised three months of cutaneous flushing, watery diarrhea, weight loss, and shortness of breath. This particular presentation prompted us to explore the possibility of carcinoid syndrome and a potential neuroendocrine tumor. A helminth infection was initially low on our differential due to the absence of eosinophilia, negative stool culture, and the atypical manifestation of flushing. We thoroughly reviewed existing literature on atypical presentations of *Strongyloides* and found no other cases associated with cutaneous flushing. *Strongyloides* is known for mimicking other diseases including Crohn’s disease and ulcerative colitis [11,12]. However, the current scenario also necessitates the consideration of *Strongyloides* in those who present with carcinoid-like symptoms.

## Conclusions

This case represents an unusual presentation of facial flushing in an already complex diagnosis of strongyloidiasis. Infection with *S. stercoralis* is difficult to prove due to the diagnostic challenges, specifically the low sensitivity of stool microscopy and variable eosinophilia. Although *Strongyloides* infection often presents with respiratory and gastrointestinal symptoms, other symptoms may lead to an unusual presentation. When attempting to diagnose strongyloidiasis, it is imperative to consider serological testing when there is a high clinical suspicion and initial stool O&P analyses are negative.
